# Macronutrient Intake and Socioeconomic Status: NIPPON DATA2010

**DOI:** 10.2188/jea.JE20170250

**Published:** 2018-03-05

**Authors:** Masaru Sakurai, Hideaki Nakagawa, Aya Kadota, Katsushi Yoshita, Yasuyuki Nakamura, Nagako Okuda, Nobuo Nishi, Yoshihiro Miyamoto, Hisatomi Arima, Takayoshi Ohkubo, Tomonori Okamura, Hirotsugu Ueshima, Akira Okayama, Katsuyuki Miura

**Affiliations:** 1 Department of Social and Environmental Medicine, Kanazawa Medical University, Ishikawa, Japan; 2 Medical Research Institute, Kanazawa Medical University, Ishikawa, Japan; 3 Center for Epidemiologic Research in Asia, Shiga University of Medical Science, Shiga, Japan; 4 Department of Public Health, Shiga University of Medical Science, Shiga, Japan; 5 Department of Food and Human Health Science, Osaka City University Graduate School of Human Life Science, Osaka, Japan; 6 Department of Food Science and Human Nutrition, Ryukoku University, Shiga, Japan; 7 Department of Health and Nutrition, University of Human Arts and Sciences, Saitama, Japan; 8 International Center for Nutrition and Information, National Institute of Health and Nutrition, National Institutes of Biomedical Innovation, Health and Nutrition, Tokyo, Japan; 9 Department of Preventive Cardiology, National Cerebral and Cardiovascular Center, Osaka, Japan; 10 Department of Preventive Medicine and Public Health, Faculty of Medicine, Fukuoka University, Fukuoka, Japan; 11 Department of Hygiene and Public Health, Teikyo University School of Medicine, Tokyo, Japan; 12 Department of Preventive Medicine and Public Health, Keio University School of Medicine, Tokyo, Japan; 13 Research Institute of Strategy for Prevention, Tokyo, Japan

**Keywords:** socioeconomic status, nutritional epidemiology, macronutrient intake, household income

## Abstract

**Background:**

This study examined the relationships among household income, other SES indicators, and macronutrient intake in a cross-sectional study of a representative Japanese population.

**Methods:**

In 2010, we established a cohort of participants in the National Health and Nutrition Survey (NHNS) from 300 randomly selected areas throughout Japan. A total of 2,637 participants (1,145 men and 1,492 women) were included in the study. Data from NHNS2010 and the Comprehensive Survey of Living Conditions 2010 (CSCL2010) were merged, and relationships among macronutrient intake and SES were evaluated. Additionally, socioeconomic factors associated with a risk of a higher carbohydrate/lower fat intake beyond dietary recommendations were evaluated.

**Results:**

Household income was positively associated with fat intake (*P* = 0.001 for men and <0.001 for women) and inversely associated with carbohydrate intake (*P* = 0.003 for men and <0.001 for women) after adjustments for age and other SES variables. Similar relationships were observed between equivalent household expenditure (EHE) and macronutrient intake; however, these relationships were weaker than those of household income. Older age was the factor most strongly associated with a high carbohydrate/low fat intake, followed by household income, EHE, education levels, and occupation type.

**Conclusions:**

Older age was the factor most strongly associated with a high carbohydrate/low fat intake, and some aspects of SES, such as household income, EHE, education levels, and occupation type, were independently associated with an imbalanced macronutrient intake. SES may affect the health status of individuals through the intake of macronutrients.

## INTRODUCTION

Diet quality is affected by socioeconomic status (SES). Recent meta-analyses indicated that high SES were associated with high dietary fiber, vitamin C, folate, beta-carotene, calcium, and iron intakes.^1^ However, relationships among macronutrient intake and socioeconomic status remain controversial.^1^ The relationships observed among socioeconomic factors and protein consumption have been positive,^2^^–^^4^ negative,^5^^,^^6^ or not significant.^7^ A consistent SES gradient has not been obtained for carbohydrate intake, and differences between SES categories were not significant^3^^,^^7^^,^^8^ or varied depending on the study.^4^^,^^5^^,^^9^^–^^13^ A consistent SES gradient has not been reported for total fat intake. Previous studies found a higher fat intake among low-SES groups^6^^,^^8^^,^^14^^,^^15^; however, an equivalent number of studies showed no significant differences.^7^^,^^8^^,^^10^^–^^12^^,^^16^^–^^18^ These differences may be attributed to country, ethnic origin, or the type of SES indicator.

In Japan, the data of the National Health and Nutrition Survey (NHNS) in 2014 indicated that a lower household income was associated with a higher intake of carbohydrates and lower intake of fat and protein.^19^ However, age and other aspects of SES, such as educational level, occupation, and living status (living alone or not), may also affect food intake, and these variables need to be taken into consideration when relationships between macronutrient intake and household income are evaluated.

A low carbohydrate diet has recently been reported to be good for body weight control in obese patients^20^ and in the treatment of diabetes mellitus.^21^ Our previous findings from NIPPON DATA80 showed that a diet high in fat and protein and low in carbohydrates was associated with a high risk of all cause death and death from cardiovascular diseases (CVD), particularly in women.^22^ These findings suggest that macronutrient intake affects health status, and, thus, factors affecting the intake of macronutrients need to be identified.

The present study was performed in order to examine the relationships among household income, other SES indicators, and macronutrient intake in a cross-sectional study of a randomly selected and representative Japanese population.

## METHODS

### Study population

In 2010, a prospective cohort study on CVD, the National Integrated Project for Prospective Observation of Non-communicable Disease and its Trends in the Aged 2010 (NIPPON DATA2010), was established.^23^ This study was performed among the participants of the NHNS in November 2010 (NHNS2010) and the Comprehensive Survey of Living Conditions in June 2010 (CSLC2010), which were conducted by the Ministry of Health, Labour and Welfare of Japan. The details of NHNS2010 and CSLC2010 have been described elsewhere.^24^^–^^29^

In November 2010, 8,815 residents aged 1 year and older from 300 randomly selected districts from throughout Japan participated in the dietary survey for NHNS2010. Among 7,229 participants aged at least 20 years, 3,873 (1,598 men and 2,275 women) had a blood test, and 2,898 (1,239 men and 1,659 women) agreed to participate in the baseline survey for NIPPON DATA2010, which also included an electrocardiographic analysis, urinalysis, and a questionnaire concerning CVD. Trained interviewers obtained informed consent before study participants enrolled. The Institutional Review Board of Shiga University of Medical Science (No. 22–29, 2010) approved this study.

Of the 2,898 participants, 91 were excluded because it was not possible to merge the data from NHNS2010 or CSLC2010 with NIPPON DATA2010 baseline data, and 170 were excluded because of missing data on employment status, length of education, marital/living status, or equivalent household expenditure (EHE). The remaining 2,637 participants (1,145 men and 1,492 women) were included in the present study.

### Nutritional survey

One-day dietary surveys with semi-weighed household food records were conducted by trained dietary interviewers for NHNS2010.^24^^,^^27^ Among nutrient intake data, total energy intake and macronutrient intake were used in the analysis. Macronutrient intake was expressed as % energy. Although energy from carbohydrate energy includes energy from alcohol (7.1 kcal per 1 g of alcohol) in the Dietary Reference Intakes for Japanese (2015),^30^ there are large differences in alcohol consumption between individuals, which cause lower calculated caloric intake (%energy) from fat and protein and higher intake from carbohydrate for heavy drinkers. In this study, we used total energy intake, excluding energy from alcohol, to evaluate macronutrient intake profile from foods. Anthropometric measurements were obtained by trained observers in NHNS2010. The height and weight of participants without shoes were measured and body mass index (BMI) was calculated as the ratio of weight (kg) to the square of height (m).

### SES

Information on SES was collected from self-administered questionnaires for NHNS2010 (number of household members, employment status, and annual household income [<2.0, 2.0–5.9, or ≥6.0 × 10^6^ yen/year]), CSLC2010 (monthly household expenditure of May 2010, the month before CSLC2010, and the type of residence [rent or own]), and NIPPON DATA2010 (length of education). The equivalent number of household members was calculated as the square root of the number of household members, and EHE was calculated as household expenditure divided by the equivalent number of household members. Socioeconomic factors were defined as follows: length of education (<12 or ≥12 years), household size (1 or ≥2), type of occupation (farmer or others), and EHE (tertiles).

### Statistical analysis

The characteristics of study participants are presented as the mean (standard deviation [SD]) or %. An analysis of variance (ANOVA) was used to compare the mean of total energy and macronutrient intake according to the degree of household income (low, middle, high, and unknown) and EHE (tertiles and unknown), while the χ^2^ test was used to compare categorical variables. Since age is known to be associated with food intake as well as economic factors, relationships were also evaluated by age groups (<40, 40–64, 65–74, and ≥75 years), and interactions between age and household income were evaluated using a two-way ANOVA. After confirming that the relationships among household income and macronutrient intake were similar throughout the age groups, we evaluated macronutrient intake according to household income in participants with a known household income/EHE (*n* = 2,337) adjusted for age and the equivalent number of household members (model 1), the type of occupation and education level (model 2), and owning a house and EHE (model 3) using analyses of covariance. Similarly, macronutrient intake according to EHE was adjusted for age and living status (model 1); the type of occupation, education level, and owning a house (model 2); and household income (model 3). Since household income was reported using a categorical variable, the equivalent number of household members was used for adjustments in the models. On the other hand, EHE itself was adjusted for the number of household members, and household size (1 [living alone] or ≥2 [not living alone]) was used for adjustments.

According to the Dietary Reference Intakes for Japanese (2015),^30^ in order to prevent lifestyle-related diseases in adults, the desirable percentage of energy intake (% energy) from carbohydrates is 50–65%, while that from fat is 20–30%. Logistic regression analyses were used to evaluate the risk of a diet that is extremely high in carbohydrates (≥65%) and low in fat (<20%) beyond the recommendations. Statistical analyses were performed using the Statistical Package for the Social Sciences (IBM SPSS statistics version 22.0; IBM Corporation, Armonk, NY, USA). A *P* value of <0.05 was considered to be significant.

## RESULTS

The mean age was 59.9 (SD, 15.5) years for men and 58.5 (SD, 15.8) years for women. The mean BMI was 23.8 (SD, 3.2) kg/m^2^ for men and 22.7 (SD, 3.5) kg/m^2^ for women. Age was associated with macronutrient intake, and older age was associated with a higher percentage intake of carbohydrates and lower percentage intake of fat (data were not tabulated).

Household income was inversely associated with age in men and women and with BMI in women (Table 1). A lower household income was associated with occupation (farmer), not owning a house, lower education levels, and lower EHE in men and women. EHE was associated with age in men and with BMI in women; however, these relationships were weaker than those with household income (Table 2). Lower EHE was associated with occupation (farmer), not owning a house, lower education levels, and lower household income in men and women.

**Table 1.** Characteristics of study participants according to the household income: NIPPON DATA2010

	Household income (×10^6^ yen/year)				*P*^a^

	Low (<2.0)	Middle (2.0–5.9)	High (≥6.0)	Unknown	
Men					
*N*	208	663	230	44	
Age, years	65.4 (12.2)	60.0 (15.8)	55.5 (14.8)	56.9 (19.2)	<0.001
Body mass index, kg/m^2^	23.9 (3.2)	23.8 (3.2)	23.7 (3.1)	24.1 (2.9)	0.842
Number of household members, *n*	2.3 (1.3)	2.8 (1.4)	3.5 (1.5)	3.4 (1.8)	<0.001
Household size, %					<0.001
1	23.1	8.3	2.2	11.4	
≥2	76.9	91.7	97.8	88.6	
Occupation, %					0.149
Farmer	11.5	6.8	7.0	9.1	
Others	88.5	93.2	93.0	90.9	
Education level, %					<0.001
≤12 years	84.6	67.3	49.6	77.3	
>12 years	15.4	32.7	50.4	22.7	
Owning a house, %					<0.001
Yes	67.8	80.4	87.8	68.2	
Equivalent household expenditure, tertile (×10^4^ yen/month), %					<0.001
High (≥15.0)	45.7	29.9	24.3	50.0	
Middle (10.5–14.9)	26.4	33.5	28.3	27.3	
Low (<10.5)	15.9	30.9	45.2	15.9	
Unknown	12.0	5.7	2.2	6.8	
Women					
*N*	320	811	292	69	
Age, years	65.4 (14.3)	58.2 (15.8)	51.6 (13.7)	58.7 (17.7)	<0.001
Body mass index, kg/m^2^	23.4 (3.8)	22.7 (3.5)	21.6 (3.2)	22.7 (3.9)	<0.001
Number of household members, *n*	2.1 (1.3)	2.9 (1.4)	3.5 (1.3)	2.9 (1.7)	<0.001
Household size, %					<0.001
1	38.8	7.3	1.4	26.1	
≥2	61.3	92.7	98.6	73.9	
Occupation, %					0.112
Farmer	5.0	2.6	2.1	4.3	
Others	95.0	97.4	97.9	95.7	
Education level, %					<0.001
≤12 years	85.9	70.2	50.0	81.2	
>12 years	14.1	29.8	50.0	18.8	
Owning a house, %					<0.001
Yes	68.4	79.8	91.4	66.7	
Equivalent household expenditure, tertile (×10^4^ yen/month), %					<0.001
High (≥15.0)	49.1	24.0	19.2	53.6	
Middle (10.5–14.9)	25.0	35.0	29.8	23.2	
Low (<10.5)	14.4	32.4	46.6	13.0	
Unknown	11.6	8.5	4.5	10.1	

Data are *n*, mean (standard deviation [SD]), or %.

^a^One-way ANOVA for continuous variables and χ^2^ test for categorical variables.

**Table 2.** Characteristics of study participants according to the equivalent household expenditure: NIPPON DATA2010

	Equivalent household expenditure				*P*^a^

	Low (<10.5)	Middle (10.5–14.9)	High (≥15.0)	Unknown	
Men					
*N*	370	312	392	71	
Age, years	58.3 (16.6)	61.1 (14.6)	60.9 (14.7)	58.7 (17.0)	0.045
Body mass index, kg/m^2^	23.7 (3.3)	23.9 (3.2)	23.8 (3.0)	24.2 (3.6)	0.623
Number of household members, *n*	3.1 (1.6)	2.8 (1.3)	2.8 (1.4)	3.1 (1.8)	0.005
Occupation, %					<0.001
Farmer	12.2	8.0	3.6	7.0	
Others	87.8	92.0	96.4	93.0	
Education level, %					<0.001
≤12 years	74.1	70.2	58.4	74.6	
>12 years	25.9	29.8	41.6	25.4	
Household size, %					0.008
1	14.6	7.4	15.1	9.9	
≥2	85.4	92.6	84.9	90.1	
Owning a house, %					<0.001
Yes	80.0	81.1	83.2	43.7	
Household income, tertile (×10^6^ yen/year), %					<0.001
High (≥6.0)	25.7	15.7	9.9	35.2	
Middle (2.0–5.9)	53.2	62.5	59.4	53.5	
Low (<2.0)	15.1	17.9	28.8	7.0	
Unknown	5.9	3.8	1.8	4.2	
Women					
*N*	441	395	530	126	
Age, years	59.7 (16.3)	58.3 (15.2)	57.7 (15.5)	58.2 (17.1)	0.258
Body mass index, kg/m^2^	22.7 (3.7)	23.0 (3.5)	22.2 (3.3)	23.5 (3.7)	0.001
Number of household members, *n*	2.9 (1.6)	2.8 (1.4)	2.8 (1.3)	3.0 (1.8)	0.181
Occupations, %					0.008
Farmer	5.4	2.3	1.9	2.4	
Others	94.6	97.7	98.1	97.6	
Education level, %					<0.001
≤12 years	77.6	71.6	63.0	69.8	
>12 years	22.4	28.4	37.0	30.2	
Household size, %					0.001
1	20.9	10.4	15.7	15.1	
≥2	79.1	89.6	84.3	84.9	
Owning a house, %					<0.001
Yes	82.3	78.7	85.3	42.1	
Household income, tertile (×10^6^ yen/year), %					<0.001
High (≥6.0)	34.9	18.0	10.9	29.4	
Middle (2.0–5.9)	44.0	62.3	57.0	54.8	
Low (<2.0)	12.7	15.9	30.2	10.3	
Unknown	8.4	3.8	1.9	5.6	

Data are *n*, mean (standard deviation [SD]), or %.

^a^One-way ANOVA for continuous variables and χ^2^ test for categorical variables.

Since age and household income were associated with each other and were also strongly associated with macronutrient intake, we evaluated the independent impacts of age and household income on macronutrient intake. Protein intake was positively associated with age (*P* < 0.001 for men and women) but was not associated with household income in men and women (*P* = 0.665 for men and *P* = 0.189 for women; data not shown). Age and household income were independently and positively associated with fat intake in men and women (*P* < 0.05 for all), and an interaction was not observed (*P* for the interaction = 0.151 for men and *P* for the interaction = 0.911 for women). Similarly, age and household income were independently and inversely associated with carbohydrate intake in men and women (*P* < 0.05 for all), and an interaction was not observed (*P* for the interaction = 0.213 for men and *P* for the interaction = 0.794 for women).

Since relationships among household income and macronutrient intake were similar in all age groups in men and women, we evaluated macronutrient intake adjusted for age and other socioeconomic factors and performed comparisons among the three groups according to household income (Table 3). Household income was positively associated with fat intake and inversely associated with carbohydrate intake after adjustments for age and the number of household members (model 1) and other SES variables (model 2). Results were similar after further adjustments for EHE (model 3). Similarly, relationships among EHE and macronutrient intake were evaluated (Table 4), and EHE was positively associated with fat and protein intakes and inversely associated with carbohydrate intake after adjustments for age and SES variables (model 1 and 2). Results were similar after further adjustments for household income; however, the relationship between EHE and fat intake was not significant in men (*P* = 0.123) or women (*P* = 0.117) (model 3).

**Table 3.** Adjusted intakes of macronutrients according to household income: NIPPON DATA2010

	Men				Women			
	
	Household income			*P*	Household income			*P*
	
	Low	Middle	High		Low	Middle	High	
Total energy intake, kcal/day								
Model 1	1,968 (39)	2,061 (21)	1,958 (36)	0.012	1,701 (27)	1,710 (16)	1,757 (27)	0.259
Model 2	1,969 (39)	2,062 (21)	1,953 (36)	0.009	1,703 (27)	1,710 (16)	1,754 (27)	0.336
Model 3	1,978 (40)	2,061 (21)	1,948 (37)	0.010	1,711 (28)	1,710 (16)	1,748 (28)	0.474
Protein, % energy								
Model 1	15.3 (0.2)	15.3 (0.1)	15.5 (0.2)	0.718	15.1 (0.2)	15.5 (0.1)	15.8 (0.2)	0.037
Model 2	15.3 (0.2)	15.3 (0.1)	15.5 (0.2)	0.748	15.0 (0.2)	15.5 (0.1)	15.9 (0.2)	0.016
Model 3	15.5 (0.3)	15.3 (0.1)	15.4 (0.2)	0.742	15.2 (0.2)	15.5 (0.1)	15.7 (0.2)	0.378
Fat, % energy								
Model 1	22.3 (0.5)	25.0 (0.3)	26.1 (0.5)	<0.001	24.6 (0.4)	26.2 (0.3)	27.6 (0.4)	<0.001
Model 2	23.2 (0.5)	25.0 (0.3)	25.9 (0.5)	0.001	24.7 (0.4)	26.2 (0.3)	27.5 (0.4)	<0.001
Model 3	23.2 (0.5)	25.0 (0.3)	25.9 (0.5)	0.001	24.9 (0.5)	26.1 (0.3)	27.3 (0.5)	0.003
Carbohydrate, % energy								
Model 1	61.5 (0.6)	59.5 (0.3)	58.2 (0.6)	0.001	60.2 (0.5)	58.2 (0.3)	56.5 (0.5)	<0.001
Model 2	61.3 (0.6)	59.5 (0.3)	58.4 (0.6)	0.003	60.1 (0.5)	58.2 (0.3)	56.5 (0.5)	<0.001
Model 3	61.1 (0.6)	59.5 (0.3)	58.6 (0.6)	0.015	59.7 (0.5)	58.3 (0.3)	56.8 (0.5)	0.002

Data are mean (standard error [SE]). Model 1, adjusted for age (20–39, 40–64, 65–74, and ≥75) and equivalent number of household members; Model 2, adjusted for age, equivalent number of household members, type of occupation (farmer, others), and education level; Model 3, adjusted for variables used in Model 2 plus owning a house and equivalent household expenditure.

**Table 4.** Adjusted intakes of macronutrients according to equivalent household expenditure: NIPPON DATA2010

	Men				Women			
	
	Equivalent household expenditure			*P*	Equivalent household expenditure			*P*
	
	Low	Middle	High		Low	Middle	High	
Total energy intake, kcal/day								
Model 1	2,000 (28)	2,038 (30)	2,029 (26)	0.624	1,696 (21)	1,711 (22)	1,741 (19)	0.266
Model 2	1,996 (28)	2,039 (30)	2,032 (27)	0.526	1,695 (21)	1,712 (22)	1,740 (19)	0.280
Model 3	1,997 (28)	2,035 (30)	2,034 (27)	0.566	1,697 (22)	1,714 (22)	1,738 (19)	0.372
Protein, % energy								
Model 1	14.9 (0.2)	15.5 (0.2)	15.8 (0.2)	0.002	15.1 (0.2)	15.4 (0.2)	15.8 (0.1)	0.001
Model 2	14.9 (0.2)	15.5 (0.2)	15.7 (0.2)	0.003	15.0 (0.2)	15.4 (0.2)	15.8 (0.1)	<0.001
Model 3	14.9 (0.2)	15.5 (0.2)	15.8 (0.2)	0.001	15.1 (0.2)	15.4 (0.2)	15.8 (0.1)	0.002
Fat, % energy								
Model 1	24.3 (0.4)	24.4 (0.4)	25.8 (0.3)	0.005	25.3 (0.3)	25.989 (0.4)	26.9 (0.3)	0.002
Model 2	24.5 (0.4)	24.4 (0.4)	25.6 (0.4)	0.028	25.4 (0.3)	25.979 (0.4)	26.8 (0.3)	0.008
Model 3	24.7 (0.4)	24.4 (0.4)	25.4 (0.4)	0.123	25.6 (0.4)	26.021 (0.4)	26.6 (0.3)	0.117
Carbohydrate, % energy								
Model 1	60.6 (0.4)	60.0 (0.5)	58.3 (0.4)	<0.001	59.5 (0.4)	58.5 (0.4)	57.1 (0.3)	<0.001
Model 2	60.4 (0.4)	60.0 (0.5)	58.5 (0.4)	0.003	59.4 (0.4)	58.5 (0.4)	57.2 (0.3)	<0.001
Model 3	60.3 (0.4)	60.0 (0.5)	58.6 (0.4)	0.017	59.2 (0.4)	58.4 (0.4)	57.4 (0.4)	0.005

Data are mean (standard error [SE]). Model 1, adjusted for age (20–39, 40–64, 65–74, ≥75) and number of household members (1, ≥2); Model 2, adjusted for age, number of household members (1, ≥2), type of occupation (farmer, others), education level, and owning a house; Model 3, adjusted for variables used in Model 2 plus household income.

We then evaluated the risk of an imbalance in the relative proportions of macronutrients, marked by extremely low fat (<20% energy) and high carbohydrate (>65% energy) beyond the desirable range proposed by the Dietary Reference Intakes for Japanese (2015) because these imbalances are commonly observed in older individuals and those with a lower household income. Age was the most strongly associated with an imbalance in macronutrient intake, followed by household income, sex, EHE, education levels, and the type of occupation (farmer), and these factors were independently associated (Table 5).

**Table 5.** Prevalence and odds ratio for high carbohydrate and low fat intakes^a^: NIPPON DATA2010

	Prevalence of participants with low fat and high carbohydrate intakes	OR	(95% confidence interval)
Sex			
Men	19.3	1.00	(reference)
Women	14.1	0.68	(0.54–0.86)
Age, years			
20–39	9.3	1.00	(reference)
40–64	13.2	1.29	(0.86–1.96)
65–74	18.4	1.46	(0.94–2.26)
≥75	28.0	2.54	(1.61–4.00)
Occupation			
Farmer	32.6	1.72	(1.12–2.62)
Others	15.5	1.00	(reference)
Education level			
≤12 years	19.2	1.50	(1.12–2.00)
>12 years	10.2	1.00	(reference)
Number of household members			
1 (living alone)	21.7	1.14	(0.82–1.58)
≥2 (not living alone)	15.7	1.00	(reference)
Owning a house			
No	14.9	1.00	(reference)
Yes	17.0	1.20	(0.87–1.66)
Household income, tertile (×10^6^ yen/year)			
High (≥6.0)	10.7	1.00	(reference)
Middle (2.0–5.9)	15.5	1.24	(0.89–1.72)
Low (<2.0)	25.2	1.88	(1.26–2.80)
Equivalent household expenditure, tertile (×10^4^ yen/month)			
High (≥15.0)	11.6	1.00	(reference)
Middle (10.5–14.9)	16.6	1.35	(1.01–1.80)
Low (<10.5)	21.0	1.54	(1.16–2.04)

OR, odds ratio.

Odds ratios were adjusted for the variables in the table using logistic regression analyses.

^a^High carbohydrate and low fat intakes were defined as carbohydrate intake >65% energy and fat intake <20% energy.

## DISCUSSION

Socioeconomic factors are reportedly associated with food intake. For example, the findings of a recent meta-analysis indicated that high SES were associated with high dietary fiber, vitamin C, folate, beta-carotene, calcium, and iron intakes.^1^ However, relationships among macronutrient intake and socioeconomic status remain controversial.^1^ Differences in relationships among macronutrient intake and socioeconomic factors may be attributed to country, ethnic origin, or the type of SES indicator. In the present analysis of a nationwide survey of a general Japanese population, some aspects of SES were associated with macronutrient intake. A lower household income was associated with a higher intake of carbohydrates and lower intake of fat, and these relationships were independent of age and other socioeconomic factors. Lower EHE was also associated with a higher intake of carbohydrates and lower intake of fat; however, these relationships were weaker than those of household income.

Differences in the results on the relationships among macronutrient intake and SES may also be due to the age of study participants. The findings of NHNS in 2014 revealed that an older age was associated with a higher intake of potatoes and fruits and lower intake of meat,^19^ which may be due to changes in food preferences with age and also a cohort effect. The difference in food intakes across age groups is one of the reasons for the high carbohydrate and low fat/protein intakes in older individuals. The findings of NHNS in 2014 showed that a lower household income was associated with higher carbohydrate and lower fat intakes after adjustments for age and household size.^19^ However, macronutrient intake and household income may vary widely between younger and older individuals, and it was unclear whether the relationships among household income and macronutrient intake were similar in different age groups. We evaluated these relationships in different age groups using a two-way ANOVA and showed that a low household income was associated with high carbohydrate and low fat intakes in all age groups (ie, household income was associated with macronutrient intake independent of age).

The Dietary Reference Intakes for Japanese (2015) indicated that the range of favorable carbohydrate and fat intakes were 50–65% energy and 20–30% energy, respectively. A diet higher in carbohydrates and lower in fat beyond this recommendation was observed in 19.3% of men and 14.1% of women. Age was most strongly associated with an imbalanced macronutrient intake, and socioeconomic factors, including household income, EHE, education levels, and type of occupation (farmer), were also independently associated with an imbalanced diet. These results indicated that not only changes in food preferences with age, but also household income and other socioeconomic factors may cause an imbalanced nutritional intake. A diet high in carbohydrates may result in a low intake of protein, which may, in turn, cause low muscle mass (ie, sarcopenia),^31^^,^^32^ and this has also been associated with a high risk of all cause death and death from CVD in Japanese individuals.^22^ Education on nutritional intake, including recommendation of a higher intake of protein and fat and lower intake of carbohydrates, in older individuals with low SES may effectively improve their health status.

However, education on nutritional intake may be insufficient to change dietary intake. A previous study from NHNS in Japan showed that the intake of some food groups, such as a high intake of cereals and low intake of vegetables, fruits, fish and shellfish, and milk, were associated with a low household income.^29^ Individuals with a low household income were unable to buy vegetables, fruits, and fish due to their high cost, and a lower intake of side dishes and higher intake of rice, which is the main starchy food in Japan, may cause an imbalanced intake of macronutrients and other nutrients.

The strength of the present study was that these data were from a nationally representative cohort, so our results may be generalized to the entire Japanese population. The present analysis has several limitations. The possibility of selection bias cannot be excluded because data from individuals who were missing answers on household income were not included in the analyses. Furthermore, the dietary intake survey for NHNS was conducted on 1 day in November. This single day may not be an appropriate representation of the habitual intake of research participants.

In conclusion, a low household income was associated with higher carbohydrate intake and lower fat intake in a general Japanese population. Older age was most strongly associated with a high carbohydrate/low fat diet, and some socioeconomic factors, such as household income, EHE, education levels, and occupation type, were also independently associated with an imbalanced macronutrient intake. SES may affect the health status of individuals through the intake of macronutrients.
